# Low Urine Uromodulin Levels are Associated With Interstitial Fibrosis and Tubular Atrophy in Native Kidney Biopsies

**DOI:** 10.1016/j.ekir.2026.106351

**Published:** 2026-02-12

**Authors:** Manav C. Parikh, Heather Thiessen Philbrook, David Hu, Jack Bitzel, Jiashu Xue, Serena DSouza, Avi Z. Rosenberg, Dennis G. Moledina, Steven G. Coca, Chirag R. Parikh, Steven Menez, Kidney Precision Medicine Project

**Affiliations:** 1 College of Arts and Sciences, University of Pennsylvania, Philadelphia, Pennsylvania, USA; 2 Division of Nephrology, Johns Hopkins University School of Medicine, Baltimore, Maryland, USA; 3 Section of Nephrology, Department of Internal Medicine, Yale School of Medicine, New Haven, Connecticut, USA; 4 Icahn School of Medicine at Mount Sinai, New York, New York, USA

**Keywords:** lateral flow devices, precision medicine, urine biomarkers

## Introduction

Interstitial fibrosis and tubular atrophy (IFTA) is a critical histological feature assessed in kidney biopsies, with its severity strongly correlating with long-term clinical outcomes, including progression to end-stage kidney disease.^1^, ^2^, ^3^ Although kidney biopsy remains the gold standard for diagnosis and evaluating specific histological features such as IFTA, noninvasive methods to assess IFTA would be highly valuable for disease management, especially among patients with absolute or relative contraindications to biopsy.^4^^,^^5^

Urine uromodulin (uUMOD) is the most abundant protein in urine produced by the thick ascending limb of the Loop of Henle and has emerged as a promising biomarker of kidney health.^6^, ^7^, ^8^, ^9^ Previous studies have demonstrated a relationship between uUMOD and the presence of IFTA on kidney tissue.^8^ However, the relationship between uUMOD across the full spectrum of the degree of IFTA is underexplored. In this study, we investigated the association between uUMOD levels and the degree of IFTA, especially at higher severity of IFTA in 2 independent cohorts.

Details on data and sample collection are provided in the Supplementary Methods.S1^,^ S2

## Results

**Figure 1:**
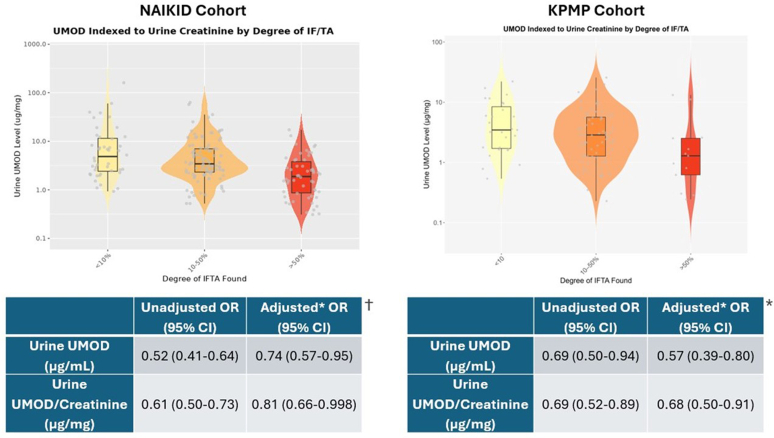
Urine UMOD (μg/mg) adjusted for creatinine by degree of IFTA level for both the NAIKiD (left) and KPMP (right) cohorts, along with odds ratios of higher degree of fibrosis (95% confidence interval) per doubling of uUMOD.†Adjusted for age (continuous), sex, estimated glomerular filtration rate (continuous), diabetes, hypertension, and urine albumin-to-creatinine at the time of biopsy. ∗Adjusted for age (< 40, 40–50, > 60 years), sex (male, female), Baseline chronic kidney disease status (estimated glomerular filtration rate < 60, ≥ 60), history of diabetes (yes/no), history of hypertension (yes/no), and urine albumin-to-creatinine, mg/g (continuous; at the time of urine UMOD measurement). IFTA, Interstitial fibrosis and tubular atrophy; UMOD, uromodulin.

A total of 112 patients (56%) in the NAIKiD cohort and 27 (24.3%) in KPMP were biopsied as inpatients, and the rest as outpatients. In the NAIKiD cohort (*n* = 200, mean age: 53.4 years, 53.5% female; Supplementary Table S1), 44 patients had < 10% IFTA, 102 had 10% to 50% IFTA, and 54 had > 50% IFTA. uUMOD concentrations showed a steady decline with increasing severity of IFTA (Table 1). In unadjusted ordinal logistic regression, doubling of uUMOD and uUMOD:Cr levels were significantly associated with lower odds of more severe IFTA (odds ratio = 0.52, 95% confidence interval: 0.41–0.64 for uUMOD; odds ratio = 0.61, 95% confidence interval: 0.50–0.73 for uUMOD:Cr). After adjusting for demographics, comorbidities, estimated glomerular filtration rate, and albuminuria, each doubling in uUMOD:Cr was associated with 19% lower odds of more severe IFTA. Similar trends were observed for uUMOD concentration alone (Figure 1).

**Table 1:** Summary statistics of adjusted and unadjusted uUMOD values around biopsy for the NAIKID and KPMP cohorts

			IFTA category			*P*-value
Cohort	Number per IFTA category	Total	< 10%	10%–50%	> 50%	
	Number per IFTA category	*N = 200*	*n = 44*	*n = 102*	*n = 54*	
NAIKID	UMOD concentration, μg/ml					
	Mean (SD)	3.86 (3.84)	5.29 (5.52)	4.21 (3.33)	2.05 (2.06)	< 0.001
	Median (min–max)	3.03 (0.146–32.3)	3.61 (0.797–32.3)	3.34 (0.180–20.8)	1.15 (0.146–10.4)	
	Urine UMOD (μg/mg)					
	Mean (SD)	7.10 (14.5)	13.0 (26.1)	6.76 (9.70)	2.93 (3.25)	< 0.001
	Median (min–max)	3.03 (0.309–161)	4.87 (0.931–161)	3.43 (0.522–63.2)	1.88 (0.309–17.2)	

	Number per IFTA category	*N = 109*	*n = 27*	*n = 69*	*n = 13*	
KPMP	UMOD concentration, μg/ml					
	Mean (SD)	2.82 (2.51)	3.50 (3.83)	2.79 (1.88)	1.56 (1.35)	0.026
	Median (min–max)	2.20 (0.141–20.7)	2.41 (0.14–20.7)	2.42 (0.170–8.97)	1.07 (0.182–4.99)	
	Urine UMOD (μg/mg)					
	Mean (SD)	4.48 (4.85)	5.76 (5.48)	4.29 (4.64)	2.85 (4.23)	0.024
	Median (min–max)	2.77 (0.23–25.7)	3.46 (0.54–22.1)	2.86 (0.23–25.7)	1.29 (0.244–13.2)	

KPMP, Kidney Precision Medicine Project Study; IFTA, Interstitial fibrosis and tubular atrophy; NAIKID Novel Approaches in Investigation of Kidney Disease Study; UMOD, uromodulin.

In the KPMP cohort (*n* = 109, 41.3% female; Supplementary Table S2), 27 patients had < 10% IFTA, 69 had 10% to 50% IFTA, and 13 had > 50% IFTA. In unadjusted ordinal logistic regression, doubling of uUMOD and uUMOD:Cr levels were significantly associated with lower odds of more severe IFTA (odds ratio = 0.69, 95% confidence interval: 0.50–0.94 for uUMOD; OR = 0.69, 95% confidence interval: 0.52–0.89 for uUMOD:Cr; Table 1). After adjustment, each doubling in uUMOD:Cr was associated with 32% lower odds of more severe IFTA (Figure 1). The AUC’s for UMOD and uMOD:Cr in predicting IFTA ≥ 50% versus < 50% ranged from 0.7 to 0.77 across both cohorts. Details on the optimal cut points and receiver operator characteristics can be found in Supplementary Table S3.

Using LFDs, we observed a comparable reduction when comparing a cut-off of < 50% fibrosis to > 50% fibrosis (Supplementary Figure S2).

## Discussion

In this study, we found a significant inverse association between uUMOD levels across a broad range of IFTA severity in 2 distinct patient cohorts. This finding expands upon previous research, highlighting that lower uUMOD levels are strongly associated with higher degrees of IFTA, particularly exceeding 50%. This can serve as a clinically meaningful cutoff with implications for overall poor prognosis and assist with clinical decision-making around kidney biopsy and immunosuppressive management. In addition, uMOD:Cr seems to have moderate discrimination ability by itself, and future studies should explore the predictive diagnostic value of uUMOD combined with other biomarkers and clinical variables in larger cohorts. The measurement of uUMOD as an indicator of the degree of IFTA highlights how noninvasive biomarkers remain promising in the development of a “liquid biopsy.”

The same inverse relationship between uUMOD and IFTA when using LFDs in a subset of the patient cohort demonstrates a clinical application of such devices to determine urine biomarker concentration and thereby degree of scarring. Therefore, this study contains empirical evidence of 1 possible approach for a “liquid biopsy.” More broadly, the LFD results demonstrate the promise of urinalysis as a reliable alternative method for estimating kidney health. The fact that a point-of-care tool’s output aligns with our study’s results using Meso Scale Discovery and “classic” urinalysis highlights the progress in creating quicker and cost-effective therapies.

Our study has several major strengths. All patients included in the final analytic cohort were well-phenotyped, with extensive electronic health record data available for analysis. In the KPMP cohort, rigorous histological adjudication was performed with 10 independent pathologists. In addition, across both cohorts, there was a broad range of IFTA values reported from pathology reports ranging from < 10% IFTA to > 90% fibrosis.

We acknowledge several key limitations in this study. The 2 cohorts differ in many aspects, including the timing of biopsy, inclusion and exclusion criteria, hospital setting, and covariates for model adjustment. These differences are likely to contribute to the discrepancies observed in the association metrics as well as cut points for the AUC. The smaller sample size of both cohorts limited the number of adjustment variables that were able to be included in the regression and AUC model. Finally, the cross-sectional nature of this study makes it difficult to make conclusions about the effect of changes in uUMOD on IFTA progression over time. Future studies involving longitudinal monitoring and the associations of uUMOD and IFTA, particularly those that use larger cohorts, will enhance its clinical utility by enabling more robust multivariate association and prediction models.

In conclusion, higher uUMOD and uUMOD:Cr were associated with lower IFTA. Prospective studies with clinically available standardized UMOD assays have the potential to be a part of routine kidney health monitoring. Future studies with larger sample sizes, more robust use of LFDs and point-of-care estimate tools, and with exploration of additional biomarkers for key histological features paired with deeper molecular phenotyping, will aid in the development of robust predictive models of kidney histology.

## Disclosure

SGC reported serving as a member of the advisory board of and owns equity in RenalytixAI and has received consulting fees from Goldfinch Bio, CHF Solutions, Quark Biopharma, Janssen Pharmaceuticals, Takeda Pharmaceuticals, and Relypsa in the past 3 years. DGM and CRP are named coinventors on a pending patent, “Methods and Systems for Diagnosis of Acute Interstitial Nephritis.” DGM and CRP are founders of the diagnostics company Predict AIN, LLC. SM reports royalties from McGraw-Hill. All the other authors declared no competing interests.

## Supplementary Material

Supplementary File (PDF) **Supplemental Methods. Supplementary References. Figure S1.** Recombinant protein standard curve for UMOD using lateral flow device. **Figure S2.** uUMOD values by binary IFTA classification measured by lateral flow device in 30 patients in the NAIKiD cohort. **Table S1.** Clinical and demographic variables at the time of biopsy for the NAIKiD study cohort. **Table S2.** Clinical and demographic variables for the KPMP study cohort. **Table S3.** AUCs for uUMOD prediction of binary IFTA outcome for NAIKiD and KPMP cohorts. STROBE Checklist. **List of the Members of the Kidney Precision Medicine Project**.

## References

[1] An Y., Xu F., Le W. (2015). Renal histologic changes and the outcome in patients with diabetic nephropathy. Nephrol Dial Transplant.

[2] Alamartine E., Sauron C., Laurent B., Sury A., Seffert A., Mariat C. (2011). The use of the Oxford classification of IgA nephropathy to predict renal survival. Clin J Am Soc Nephrol.

[3] Wilson P.C., Kashgarian M., Moeckel G. (2018). Interstitial inflammation and interstitial fibrosis and tubular atrophy predict renal survival in lupus nephritis. Clin Kidney J.

[4] Poggio E.D., McClelland R.L., Blank K.N. (2020). Systematic review and meta-analysis of native kidney biopsy complications. Clin J Am Soc Nephrol.

[5] Schnuelle P. (2023). Renal biopsy for diagnosis in kidney disease: indication, technique, and safety. J Clin Med.

[6] Micanovic R., LaFavers K., Garimella P.S., Wu X.R., El-Achkar T.M. (2019). Uromodulin (tamm-Horsfall protein): guardian of urinary and systemic homeostasis. Nephrol Dial Transplant.

[7] El-Achkar T.M., Wu X.R. (2012). Uromodulin in kidney injury: an instigator, bystander, or protector?. Am J Kidney Dis.

[8] Melchinger H., Calderon-Gutierrez F., Obeid W. (2022). Urine uromodulin as a biomarker of kidney tubulointerstitial fibrosis. Clin J Am Soc Nephrol.

[9] Devuyst O., Olinger E., Rampoldi L. (2017). Uromodulin: from physiology to rare and complex kidney disorders. Nat Rev Nephrol.

