# Supplementary material for: Low Urine Uromodulin Levels are Associated With Interstitial Fibrosis and Tubular Atrophy in Native Kidney Biopsies
Source: Kidney Int Rep. 2026 Feb 12;11(4):106351. doi: 10.1016/j.ekir.2026.106351 (PMC13010425; doi:10.1016/j.ekir.2026.106351)
Supplement: Supplementary File (PDF) — Supplemental Methods. Supplementary References. Figure S1. Recombinant protein standard curve for UMOD using lateral flow device. Figure S2. uUMOD values by binary IFTA classification measured by lateral flow device in 30 patients in the NAIKiD cohort. Table S1. Clinical and demographic variables at the time of biopsy for the NAIKiD study cohort. Table S2. Clinical and demographic variables for the KPMP study cohort. Table S3. AUCs for uUMOD prediction of binary IFTA outcome for NAIKiD and KPMP cohorts. STROBE Checklist. List of the Members of the Kidney Precision Medicine Project. [file mmc1.pdf]

SUPPLEMENTAL MATERIAL

Lower Urine Uromodulin Levels Associated with Interstitial Fibrosis and Tubular Atrophy in Native Kidney Biopsies

Manav C. Parikh;[1,2] Heather Thiessen Philbrook, MMath;[2] David Hu, MS;[2] Jack Bitzel, BS;[2] Jiashu Xue, MS;[2] Serena D Souza, Ph.D.;[2] Avi Z. Rosenberg;[2] Dennis G. Moledina, MD, PhD;[3] Steven G. Coca DO, MS;[4] Chirag R. Parikh, MD, PhD;[2] Steven Menez, MD, MHS[2] and the Kidney Precision Medicine Project

[1]University of Pennsylvania, Philadelphia, PA, USA

[2]Division of Nephrology, Johns Hopkins University School of Medicine, Baltimore, Maryland

[3]Section of Nephrology, Department of Internal Medicine, Yale University School of Medicine, New Haven, Connecticut

[4]Division of Nephrology, Icahn School of Medicine at Mount Sinai, New York, NY

We leveraged data and biosamples from 200 adult patients undergoing clinically indicative native kidney biopsies between September 2020 and February 2023 at the Johns Hopkins Hospital enrolled to the Novel Approaches in Investigation of Kidney Disease (NAIKiD) Study, in which patients scheduled for clinically indicated native kidney biopsies contributed blood, urine, and kidney tissue toward building a kidney biopsy registry. In addition, we analyzed data from 109 patients across 13 sites in the Kidney Precision Medicine Project Study (KPMP).[S1] The KPMP is a multicenter prospective cohort study of people with chronic kidney disease or acute kidney injury who undergo a protocol kidney biopsy at study entry as part of the KPMP consortium, in addition to participants enrolled as healthy controls, and patients classified as being resilient with diabetes mellitus. The degree of IFTA present on kidney biopsy was categorized into 3 groups as follows: minimal or absent (< 10% IFTA), moderate (10%–50% IFTA), and severe (> 50% IFTA) based on clinical convention and prior literature.[1–3] Ordinal logistic regression was used to evaluate the association between uUMOD level and degree of IFTA in both univariate and models adjusted for age, sex, diabetes, hypertension, estimated glomerular filtration rate, and urine albumin-to-creatinine (Cr) ratio at the time of biopsy. We evaluated the predictive performance of uUMOD using the area under the receiver operator characteristic curve (AUC) for IFTA > 50% versus ≤ 50%. To account for developing and evaluating the model in the same cohort, a bootstrap procedure of 1000 samples was used to estimate the optimism-corrected AUC. Receiver operator curve characteristics (such as sensitivity and specificity) were reported for the Youden Index. uUMOD was measured in 30 patients using lateral flow devices (LFDs) in the NAIKiD cohort, developed specifically for the detection of uUMOD.[S2]

Supplementary Methods

uUMOD was measured using a multiplex assay on the Meso Scale Discovery (MSD) platform, with standardized protocols and reagents across both cohorts in our laboratory to minimize measurement bias. Creatinine in the urine was measured by Randox Daytona® autoanalyzer. Laboratory personnel were blinded to clinical and histological data.

Sex as a biological variable:

Sex was included as a biological variable. Our study examined both male and female humans, and sex was a variable adjusted for in the ordinal regression calculation. In both the NAIKiD and KPMP cohorts, the distribution of males and females across the three degrees of IFTA was significantly different. **(Supplementary Tables 1,2)**

Statistics:

The Kruskal-Wallis Test was used to compare different IFTA severity groups across the continuous variables uUMOD, uUMOD indexed to urine creatinine (uUMOD:Cr), eGFR, albumin: creatinine ratio (ACR) based on degree of IFTA. A 3-way ANOVA test was used to calculate similar differences of age at the time of biopsy, and a chi-square test was used for significant hypertension and diabetes differences in the three groups. The proportional odds assumption was verified graphically. All analyses were conducted in R.

Study Approval:

All studies included in this manuscript received approval from their respective institutional review boards at each participating center (IRB Number: IRB00282082).

Data Availability

Data for the KPMP study is publicly available through the KPMP Atlas or after request to the KPMP consortium with a Data Use Agreement. The NAIKID study is from a consented observational research study which is not publicly available but may be available upon

reasonable request to the corresponding author with the completion of study regulatory requirements.

NAIKiD Study

The Novel Approaches in the Investigation of Kidney Disease was started in September of 2020 and aims to enroll both inpatients and outpatients scheduled for clinically indicated native kidney biopsies at the Johns Hopkins Hospital to contribute blood, urine, and kidney tissue towards building a kidney biopsy registry. All adult patients scheduled for native kidney biopsy are screened for eligibility. Study exclusion criteria include age <18 years old and inability to provide written informed consent.

For research kidney tissue collection, patients are asked if they are willing to consent to an extra core of kidney tissue, unless they meet the following exclusion criteria: hemoglobin level < 8 g/dL, platelet count < 75,000, use of aspirin within 5 days prior biopsy, use of non-steroidal anti-inflammatory drugs within 48 hours prior to biopsy, confirmed or suspected pregnancy, inability to withdraw systemic anticoagulation at least 24 hours before or 48 hours after biopsy, INR > 1.4, severe iodine allergy, Jehovah's Witness or otherwise unwilling to receive a blood transfusion, or any other concerns by the biopsy operator where an extra core of kidney tissue cannot be safely obtained. For patients who do not consent to, or are not eligible for, an extra core of kidney tissue, residual tissue is obtained after clinical processing by the division of kidney pathology. Patients with hemoglobin < 7 g/dL are excluded from consideration for blood sample collection.

In addition to active data collection through patient questionnaires taken at the time of study enrollment, patients enrolled into the NAIKiD study are additionally asked if they consent passive data collection within the Johns Hopkins Epic electronic medical record (EMR) system, as well as external EMR systems through the Chesapeake Regional Information System for our Patients (CRISP) regional health information exchange.

Patient demographic and clinical data were collected through a combination of active data collection obtained at the time of NAIKiD study enrollment, as well as through passive data collection through the Johns Hopkins Kidney Precision Medicine Center of Excellence (KPMCOE). The estimated glomerular filtration rate (eGFR) was calculated using the race-free CKD-EPI equation. The degree of IFTA was obtained from pathology reports.

The urine samples for NAIKID were collected within 24-48 hours before or after biopsy.

KPMP Study

The detailed inclusion and exclusion criteria for AKI and CKD participants are defined in the general KPMP study rationale.[9] The degree of IFTA was defined as the average of the Interstitial Fibrosis (%) and Tubular Atrophy (%) which was scored by the KPMP Pathology TIV Descriptor Scoring Task Force. The median time between biopsy and urine sampling was median[IQR] of 2[1-15] days.

Uromodulin Assay Details

The uUMOD MSD detection ranges for uUMOD were 0.01 μg/mL to 100 μg/mL. The average inter-assay and intra-assay coefficients of variation were 14.2% and 4.8%, respectively. Urine creatinine was measured using the Randox RX Daytona clinical chemistry analyzer (Randox, UK). Both biomarkers were measured using the same protocol and batch of reagents for both cohorts in the JHU biomarker laboratory.

Lateral Flow Devices

Lateral Flow Devices (LFD) are a Point-Of-Care tool that uses specific antibodies to determine urine biomarker concentration.

Recombinant protein standard curve generation and measurement of urine samples using Lateral Flow Devices (LFD)

A recombinant protein standard curve (0-4 μg /mL) was generated for UMOD (Raybiotech, USA) using the lateral flow device (Mologic D/B/A Global Access Diagnostics: GADx), USA). Three

drops of the UMOD standard (prepared and filled in a custom-made diluent tube) were added to the device and incubated for 20 minutes on a flat surface. The results were read using a hand-held RDS-2500 reader (DETEKT, USA) in the form of control (CL) and test line (TL) intensity. A ratio of the TL/CL intensity was computed to analyze the results (Supplemental Figure 1).

For the urine measurement, 30 patients from the NAIKiD cohort across all severities of fibrosis were selected for LFD measurement. The centrifuged urine samples were diluted (1:20) and added to the custom-made diluent tube. The diluent tube was inverted 5 times. Three drops of the diluted sample were added to the LFD and incubated for 20 minutes. The results were read using a handheld RDS-2500 reader (DETEKT, USA) in the form of a ratio of test line (TL) to control line (CL) intensity (**Supplemental Figure 1**).

<u>Supplemental Table 1</u>: Clinical and Demographic Variables at the Time of Biopsy for the NAIKiD Study Cohort

		IFTA Level			
	Total (N=200)	<10% (N=44)	10-50% (N=102)	>50% (N=54)	P-value
**Age at Biopsy**					
Mean (SD)	53.4 (16.6)	46.2 (17.2)	55.1 (16.3)	56.1 (15.0)	0.017
Median [Min, Max]	53.6 [19.6, 84.1]	43.7 [19.6, 78.4]	57.5 [20.8, 84.1]	56.4 [21.4, 78.0]	
**Gender**					
Female	107 (53.5%)	31 (70.5%)	55 (53.9%)	21 (38.9%)	0.021
Male	93 (46.5%)	13 (29.5%)	47 (46.1%)	33 (61.1%)	
**Hypertension**					
No	87 (43.5%)	32 (72.7%)	41 (40.2%)	14 (25.9%)	<0.001
Yes	113 (56.5%)	12 (27.3%)	61 (59.8%)	40 (74.1%)	
**Diabetes**					
No	161 (80.5%)	41 (93.2%)	83 (81.4%)	37 (68.5%)	0.023
Yes	39 (19.5%)	3 (6.8%)	19 (18.6%)	17 (31.5%)	
**eGFR (mL/min/1.73 m²)**					
Mean (SD)	53.7 (38.8)	86.4 (43.7)	54.4 (33.1)	25.9 (18.4)	<0.001
Median [Min, Max]	41.6 [5.15, 169]	97.0 [11.5, 169]	48.0 [8.27, 128]	20.0 [5.15, 88.3]	
**Albumin: Creatinine Ratio**					
Mean (SD)	1670 (2230)	1220 (2550)	1430 (1840)	2490 (2430)	<0.001
Median [Min, Max]	764 [3.54, 13900]	286 [3.54, 13900]	535 [9.89, 9660]	1800 [17.3, 9900]	

*Urine Albumin and Creatinine used to calculate ACR in mg/dL

Supplemental Table 2: Clinical and Demographic Variables for the KPMP Study Cohort

		Total (N=109)	IFTA Category <10% (N=27)	10-50% (N=69)	>50% (N=13)	p value
Age	<40	20 (18.3%)	8 (29.6%)	7 (10.1%)	5 (38.5%)	0.006
	40-59	33 (30.3%)	4 (14.8%)	23 (33.3%)	6 (46.2%)	
	>=60	56 (51.4%)	15 (55.6%)	39 (56.5%)	2 (15.4%)	
Sex	Female	45 (41.3%)	13 (48.1%)	31 (44.9%)	1 (7.7%)	0.031
	Male	64 (58.7%)	14 (51.9%)	38 (55.1%)	12 (92.3%)	
History of Hypertension	No	22 (20.2%)	11 (40.7%)	11 (15.9%)	0 (0.0%)	0.004
	Yes	87 (79.8%)	16 (59.3%)	58 (84.1%)	13 (100.0%)	
History of Diabetes	No	31 (28.4%)	14 (51.9%)	13 (18.8%)	4 (30.8%)	0.005
	Yes	78 (71.6%)	13 (48.1%)	56 (81.2%)	9 (69.2%)	
Baseline CKD	No	49 (45.0%)	15 (55.6%)	28 (40.6%)	6 (46.2%)	0.413
	Yes	60 (55.0%)	12 (44.4%)	41 (59.4%)	7 (53.8%)	
UACR (mg/g)	Median (Min, Max)	119 (7, 7253)	54 (8, 1771)	162 (7, 7253)	2225 (17, 6236)	0.003

*UACR (mg/g) = Urine Albumin to Creatinine Ratio measured at the same time as Urine UMOD.

*Baseline CKD is defined as baseline eGFR <60 ml/min per 1.73 m^2

<u>Supplemental Table 3:</u> AUCs for uUMOD Prediction of Binary IFTA Outcome for NAIKiD and KPMP Cohort

	**Outcome: IFTA>50% vs. <50%**							
	**NAIKID (n=200)** Events: 54 (27%)				**KPMP (n=109)** Events: 13 (12%)			
**Model**	**AUC* (95% CI)**	**Threshold†**	**Sensitivity**	**Specificity**	**AUC* (95% CI)**	**Threshold†**	**Sensitivity**	**Specificity**
**Urine UMOD (ug/mL)**	0.771 *(0.693, 0.850)*	0.106	0.769	0.667	0.733 *(0.592,0.883)*	0.346	0.667	0.788
**Urine Creatinine Corrected UMOD (ug/mg)**	0.727 *(0.647, 0.807)*	0.098	0.846	0.573	0.701 *(0.54,0.863)*	0.359	0.537	0.836

*AUC are optimism corrected using a bootstrap procedure (1000 bootstrap samples)

†Threshold and ROC characteristics are reported for Youden index.

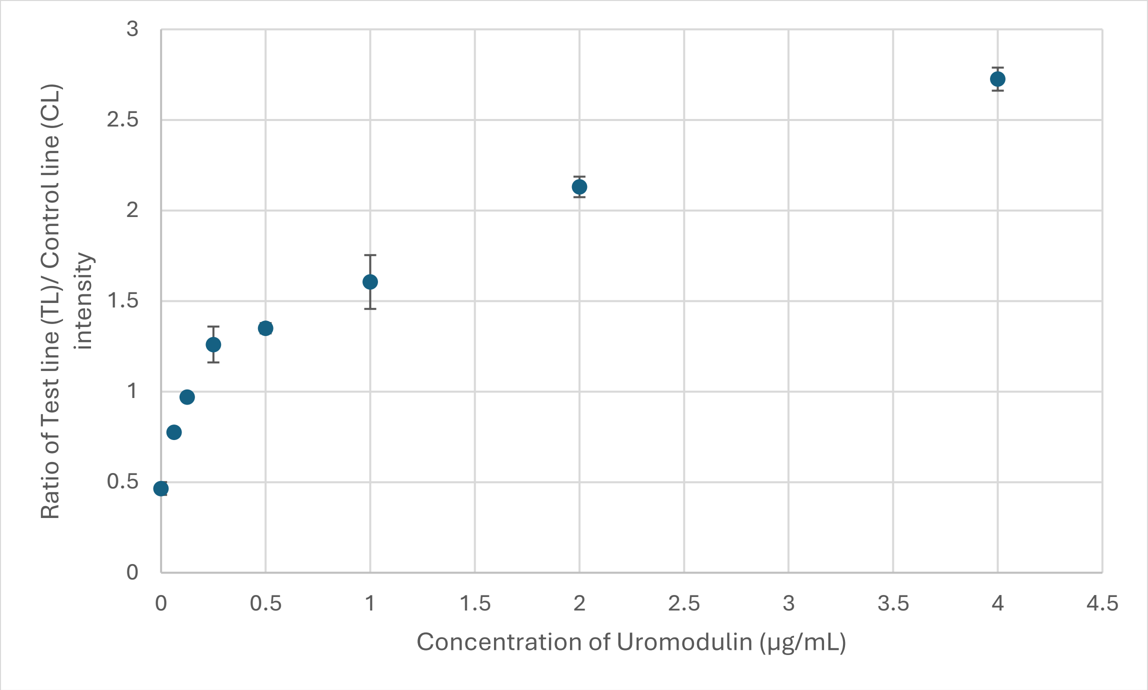

<u>Supplemental Figure 1:</u> Recombinant Protein Standard Curve for UMOD Using Lateral Flow Device

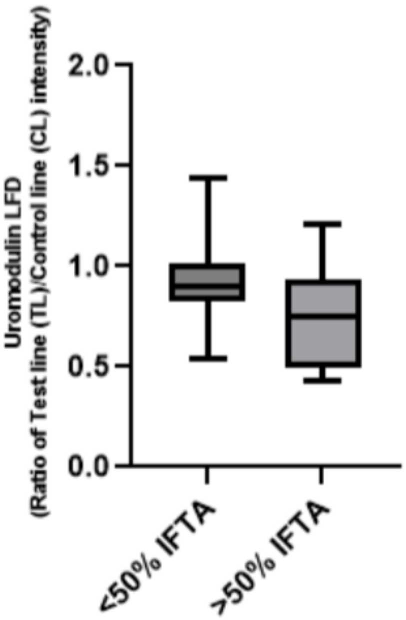

Figure 2: Box plot for UMOD measurements by LFD (2 categories)

Mann Whitney test: p values (0.0474)

Supplemental Figure 2: uUMOD Values by Binary IFTA Classification Measured by Lateral Flow Device in 30 Patients in the NAIKiD Cohort

Supplementary References

S1. de Boer IH, Alpers CE, Azeloglu EU*, et al.* Rationale and design of the Kidney Precision Medicine Project. *Kidney International* 2021; **99:** 498-510.

S2. D Souza S, Obeid W, Hernandez J*, et al*. The development of lateral flow devices for urinary biomarkers to assess kidney health. *Scientific Reports* 2024; **14:** 8516.

STROBE Checklist

	Item No.	Recommendation	Page No.	Relevant text from manuscript
**Title and abstract**	1	(*a*) Indicate the study's design with a commonly used term in the title or the abstract	1	
		(*b*) Provide in the abstract an informative and balanced summary of what was done and what was found	NA	
**Introduction**				
Background/rationale	2	Explain the scientific background and rationale for the investigation being reported	2	
Objectives	3	State specific objectives, including any prespecified hypotheses	2	
**Methods**				
Study design	4	Present key elements of study design early in the paper	2	
Setting	5	Describe the setting, locations, and relevant dates, including periods of recruitment, exposure, follow-up, and data collection	Supplemental Methods	
Participants	6	(*a*) *Cohort study*—Give the eligibility criteria, and the sources and methods of selection of participants. Describe methods of follow-up<br>*Case-control study*—Give the eligibility criteria, and the sources and methods of case ascertainment and control selection. Give the rationale for the choice of cases and controls<br>*Cross-sectional study*—Give the eligibility criteria, and the sources and methods of selection of participants	Supplemental Methods	
		(*b*) *Cohort study*—For matched studies, give matching criteria and number of exposed and unexposed<br>*Case-control study*—For matched studies, give matching criteria and the number of controls per case		
Variables	7	Clearly define all outcomes, exposures, predictors, potential confounders, and effect modifiers. Give diagnostic criteria, if applicable	2	
Data sources/ measurement	8*	For each variable of interest, give sources of data and details of methods of assessment (measurement). Describe comparability of assessment methods if there is more than one group	Supplemental Methods	
Bias	9	Describe any efforts to address potential sources of bias	Supplemental Methods	

Study size	10	Explain how the study size was arrived at	Supplemental Methods

Continued on next page

Quantitative variables	11	Explain how quantitative variables were handled in the analyses. If applicable, describe which groupings were chosen and why	2,3
Statistical methods	12	(*a*) Describe all statistical methods, including those used to control for confounding	2,3
		(*b*) Describe any methods used to examine subgroups and interactions	2,3
		(*c*) Explain how missing data were addressed	NA
		(*d*) *Cohort study*—If applicable, explain how loss to follow-up was addressed<br>*Case-control study*—If applicable, explain how matching of cases and controls was addressed<br>*Cross-sectional study*—If applicable, describe analytical methods taking account of sampling strategy	NA
		(*e*) Describe any sensitivity analyses	3, Supplementary Material
**Results**			
Participants	13*	(a) Report numbers of individuals at each stage of study—eg numbers potentially eligible, examined for eligibility, confirmed eligible, included in the study, completing follow-up, and analysed	4
		(b) Give reasons for non-participation at each stage	NA
		(c) Consider use of a flow diagram	NA
Descriptive data	14*	(a) Give characteristics of study participants (eg demographic, clinical, social) and information on exposures and potential confounders	4
		(b) Indicate number of participants with missing data for each variable of interest	NA
		(c) *Cohort study*—Summarise follow-up time (eg, average and total amount)	NA
Outcome data	15*	*Cohort study*—Report numbers of outcome events or summary measures over time	
		Case-control study—Report numbers in each exposure category, or summary measures of exposure	
		Cross-sectional study—Report numbers of outcome events or summary measures	4
Main results	16	(*a*) Give unadjusted estimates and, if applicable, confounder-adjusted estimates and their precision (eg, 95% confidence interval). Make clear which confounders were adjusted for and why they were included	4
		(*b*) Report category boundaries when continuous variables were categorized	4
		(*c*) If relevant, consider translating estimates of relative risk into absolute risk for a meaningful time period	NA

Continued on next page

Other analyses	17	Report other analyses done—eg analyses of subgroups and interactions, and sensitivity analyses	4, Supplementary Material
**Discussion**			
Key results	18	Summarise key results with reference to study objectives	5
Limitations	19	Discuss limitations of the study, considering sources of potential bias or imprecision. Discuss both direction and magnitude of any potential bias	5,6
Interpretation	20	Give a cautious overall interpretation of results considering objectives, limitations, multiplicity of analyses, results from similar studies, and other relevant evidence	5,6
Generalisability	21	Discuss the generalisability (external validity) of the study results	5,6
**Other information**			
Funding	22	Give the source of funding and the role of the funders for the present study and, if applicable, for the original study on which the present article is based	7

*Give information separately for cases and controls in case-control studies and, if applicable, for exposed and unexposed groups in cohort and cross-sectional studies.

Note: An Explanation and Elaboration article discusses each checklist item and gives methodological background and published examples of transparent reporting. The STROBE checklist is best used in conjunction with this article (freely available on the Web sites of PLoS Medicine at http://www.plosmedicine.org/, Annals of Internal Medicine at http://www.annals.org/, and Epidemiology at http://www.epidem.com/). Information on the STROBE Initiative is available at www.strobe-statement.org.

Name	Email	Institution	Role
Oyedele A. Adeyi	adeyio@umn.edu	University of Minnesota	Pathologist
Lun Ai	ail@ebi.ac.uk	European Molecular Biology Laboratory	Fellow
Sheelashree Ajakkala Narayana	sheelashree.ajakkalanarayana@mountsinai.org	Mount Sinai	Principal Investigator,Fellow
Fadhl Alakwaa	alakwaaf@umich.edu	University of Michigan	Co-Investigator
Theodore Alexandrov	theodore.alexandrov@embl.de	European Molecular Biology Laboratory	Co-Investigator
Jamie L Allen	jamie.l.allen@vanderbilt.edu	Vanderbilt University	Study staff
CE Alpers	calp@uw.edu	University of Washington	Pathologist
Alison Bunio Alvear	bunio001@umn.edu	University of Minnesota	Study staff
Akhil Ambekar	aa647@duke.edu	Duke University	Fellow
Joed Ancheta	janche3@uic.edu	University of Illinois, Chicago	Research Coordinator
Christopher R Anderton	christopher.anderton@pnnl.gov	Pacific Northwest National Laboratory	Principal Investigator
Makayla Andre	mandre6@joslin.harvard.edu	Joslin Diabetes Center	Research Coordinator
Sophia A. Angus	sophia.angus@joslin.harvard.edu	Joslin Diabetes Center	Research Coordinator
Kavya Anjani	Kavya.Anjani@ucsf.edu	University of California San Francisco	Other
Francesca Annese	francean@med.umich.edu	University of Michigan	Other
Paul S. Appelbaum, MD	psa21@columbia.edu	Columbia University	Other
Joseph Ardayfio	joseph.ardayfio@gmail.com	Other	Patient Partner,Other
Tanima Arora	Tanima.arora@yale.edu	Yale University	Other
Heather K. Ascani	ascanihk@med.umich.edu	University of Michigan	Other
Mahla Asghari	masghari@iu.edu	Indiana University	Other
Tarek M. El-Achkar	telachka@iu.edu	Indiana University	Principal Investigator
Mohamed G. Atta	matta1@jhmi.edu	Johns Hopkins University	Co-Investigator,Biopsy Operator
Mark P. Aulisio	Mpa5@case.edu	Case Western Reserve University	Co-Investigator
Stephanie J. Aw	stephaw@bu.edu	Boston Medical Center	Other
Evren U Azeloglu	evren.azeloglu@mssm.edu	Mount Sinai	Co-Investigator
Mona Babaie	monam2@uw.edu	University of Washington	Study staff
Cathy A Bagne	pinth001@umn.edu	University of Minnesota	Research Coordinator
Olivia Balderes	ob2214@cumc.columbia.edu	Columbia University	Other
Ulysses G. J. Balis	ulysses@med.umich.edu	University of Michigan	Co-Investigator,Pathologist
Jonathan Barasch	jmb4@columbia.edu	Columbia University	Principal Investigator
Mitali Barik	mbarik1@jhu.edu	Johns Hopkins University	Research Coordinator
Laura Barisoni	laura.barisoni@duke.edu	Duke University	Co-Investigator,Pathologist
Andrew J. Barkmeier	barkmeier.andrew@mayo.edu	Mayo Clinic	Co-Investigator

Daria Barwinska	daria.barwinska@gmail.com	Indiana University	Study staff
Jeannine Basta	jbasta@wustl.edu	Washington University St. Louis	Co-Investigator
Jack Bebiak	jackb@gspire.org	Other	Patient Partner,Other
Laurence H Beck Jr	Laurence.Beck@bmc.org	Boston Medical Center	Co-Investigator
Jerica M. Berge, PhD, MPH, LMFT, CFLE	jberge@umn.edu	University of Minnesota	Co-Investigator
Ashley C Berglund	bergla@uw.edu	University of Washington	Study staff
Lauren Bernard	lbernar9@jhu.edu	Johns Hopkins University	Other
Brooke Berry	bberry3@uw.edu	University of Washington	Project Manager
David H. Beyda, MD	dbeyda@arizona.edu	University of Arizona	Co-Investigator
Jini Ashok Bhanushali	jibhanu@iu.edu	Indiana University Bloomington	Project Manager
Markus Bitzer MD	markusbi@umich.edu	University of Michigan	Co-Investigator
Petter Bjornstad	pettermb@uw.edu	University of Washington	Principal Investigator
Victoria M. Blanc	vmb@med.umich.edu	University of Michigan	Other
Kristina N Blank	blankk@uw.edu	University of Washington	Project Manager
Sharon B Bledsoe	sbledsoe@iu.edu	Indiana University	Biopsy Operator
Steve Bogen	sbogen@bostoncellstandards.com	Other	Other
Andrew S. Bomback	asb68@cumc.columbia.edu	Columbia University	Principal Investigator
Nikole Bonevich	bonevicn@med.umich.edu	University of Michigan	Project Manager,Other
Samuel Border	samuel.border@medicine.ufl.edu	University of Florida	Study staff
Katy Börner	katy@indiana.edu	Indiana University Bloomington	Other
William S. Bowen	bilbowen@iu.edu	Indiana University	Study staff
Charlotte Boys	charlotte.boys@bioquant.uni-heidelberg.de	Heidelberg University	Other
Erika R Bracamonte	erikab@arizona.edu	University of Arizona	Pathologist
Frank C. Brosius	fbrosius@arizona.edu	University of Arizona	Principal Investigator
Keith D. Brown	kdb12@icloud.com	University of Washington	Patient Partner
Liam Brown	liam.brown@yale.edu	Yale University	Research Coordinator
Lihong Bu	bu.lihong@mayo.edu	Mayo Clinic	Pathologist
Tifanny Budiman	tifanny.budiman@yale.edu	Yale University	Study staff
Andreas Bueckle	abueckle@iu.edu	Indiana University	Other
Bui, JT or Bui, James T	jtbui@uic.edu	University of Illinois, Chicago	Co-Investigator,Biopsy Operator
Ashley R Burg	ashley.burg@cchmc.org	Other	Research Coordinator,Study staff
Adam Burgess	burgessae@upmc.edu	University of Pittsburgh	Research Coordinator
Lakeshia Bush, RN, BSN	bushl3@ccf.org	Cleveland Clinic	Research Coordinator
William S. Bush	wsb36@case.edu	Case Western Reserve University	Co-Investigator

Erin J Buth	ebuth@uw.edu	University of Washington	Study staff
Qi Cai	qi.cai@utsouthwestern.edu	University of Texas Southwestern	Pathologist
Marie Florence Calixte	Marie.Calixte@bmc.org	Boston Medical Center	Research Coordinator
Tashas Cameron-Wheeler	tcamero@ad.unc.edu	University of North Carolina	Research Coordinator
Kirk N Campbell	kirk.campbell@mssm.edu	Mount Sinai	Principal Investigator
Taneisha Campbell	tcampbell@giftoflifemichigan.org	Other	Patient Partner,Other
Catherine Campbell	Cec5479@me.com	University of Texas Southwestern	Patient Partner
Baltazar Campos, MPH	bcampos9@arizona.edu	University of Arizona	Research Coordinator
Pietro A. Canetta	pac2004@cumc.columbia.edu	Columbia University	Other
Lloyd G Cantley	lloyd.cantley@yale.edu	Yale University	Principal Investigator,Co-Investigator
M. Luiza Caramori	caram001@umn.edu	University of Minnesota	Principal Investigator
Eunice Carmona-Powell	ecarmo1@uic.edu	University of Illinois, Chicago	Research Coordinator
Jonas M Carson	jcarson@uw.edu	University of Washington	Study staff
Gek Cher Chan	gek_cher_chan@nuhs.edu.sg	National University of Singapore	Co-Investigator
Lili Chan	lili.chan@mountsinai.org	Mount Sinai	Biopsy Operator
Jia-Yun Chen	jia-yun_chen@hms.harvard.edu	Harvard University	Co-Investigator
Sarah W Chen	sarah.chen@joslin.harvard.edu	Joslin Diabetes Center	Research Coordinator
Xi Chen	xichensf@gmail.com	Princeton University	Other
Yijiang Chen	yxc627@case.edu	Emory	Co-Investigator
Ying-Hua Cheng	yicheng@iu.edu	Indiana University	Co-Investigator
Maria Chilo Bejarano	mchilob1@jh.edu	Johns Hopkins University	Research Coordinator
Choudhary Moaz	moaz.choudhary@utsouthwestern.edu	University of Texas Southwestern	Co-Investigator,Biopsy Operator
James G. Cimino	jim.cimino@ucsf.edu	University of California San Francisco	Research Coordinator
Steven G. Coca	steven.coca@mssm.edu	Mount Sinai	Principal Investigator
Thomas M Coffman	tcoffman@duke.edu	Duke University	Principal Investigator
Alyson Coleman	colem212@umn.edu	University of Minnesota	Project Manager
Madeline E. Colley	madeline.colley@vanderbilt.edu	Vanderbilt University	Study staff
Mary M. Collie	Mary_Collie@med.unc.edu	University of North Carolina	Research Coordinator
Mia R. Colona	Mia.Colona@bmc.org	Brigham and Women's Hospital	Other
Kristine Conlon	kristyconlon@wustl.edu	Washington University St. Louis	Research Coordinator,Patient Partner

Ninive Conser, MLS (ASCP)cm, MPH	costan@med.umich.edu	University of Michigan	Study staff
Leslie Cooperman	COOPERL2@ccf.org	Cleveland Clinic	Other
Celia P. Corona-Villalobos	pamelacorona@jhmi.edu	Johns Hopkins University	Project Manager
Dana C. Crawford	dana.crawford@case.edu	Case Western Reserve University	Co-Investigator
Nathan Creger	cregern@med.umich.edu	University of Michigan	Other
Yarieli Cuevas-Rios	cuevasyarieli@gmail.com	Vanderbilt University	Patient Partner
Vivette . D'Agati	vdd1@columbia.edu	Columbia University	Pathologist
Donna D'Souza	ddsouza@umn.edu	University of Minnesota	Biopsy Operator
Pierre C. Dagher	Pdaghe2@iu.edu	Indiana University	Principal Investigator
Ian H. de Boer	deboer@uw.edu	University of Washington	Principal Investigator,Co-Investigator
de Caestecker M.P.	mark.de.caestecker@vumc.org	Vanderbilt University	Co-Investigator
Marina de Cos	marina.decosgomez@mssm.edu	Mount Sinai	Fellow
Joana P. Gonçalves	joana.goncalves@tudelft.nl	Other	Co-Investigator
Matthew Dekker	mdekker@uw.edu	University of Washington	Study staff
Dawit Demeke	ddemeke@umich.edu	University of Michigan	Pathologist,Fellow
Ruining Deng	r.deng@vanderbilt.edu	Vanderbilt University	Study staff
Aleksandar Denic	denic.aleksandar@mayo.edu	Mayo Clinic	Co-Investigator
Austin Derma	derma1@arizona.edu	University of Arizona	Research Coordinator
Ashveena L Dighe	ashveena.dighe@mssm.edu	Mount Sinai	Project Manager
Yanli Ding	ding0382@umn.edu	University of Minnesota	Pathologist
Katerina V. Djambazova	katerina.v.djambazova@vanderbilt.edu	Vanderbilt University	Study staff
Isabel Donohoe	isabel.donohoe@joslin.harvard.edu	Joslin Diabetes Center	Other
Frederick Dowd	dowdf@uw.edu	University of Washington	Study staff
Drawz PE	draw0003@umn.edu	University of Minnesota	Co-Investigator
Martin Dufresne	martin.dufresne@vanderbilt.edu	Vanderbilt University	Study staff
Rachel Dull	roram@umich.edu	University of Michigan	Other
Kenneth W. Dunn	kwdunn@iu.edu	Indiana University	Co-Investigator
Daniel Damian Duran	damianduran@arizona.edu	University of Arizona	Research Coordinator
Michael T Eadon	meadon@iu.edu	Indiana University	Principal Investigator
Sean Eddy	seaneddy@med.umich.edu	University of Michigan	Study staff
Michele M Elder	mie1@pitt.edu	University of Pittsburgh	Project Manager
Lorraine Evo-Ortega	Lorraine.Evo-Ortega@mssm.edu	Mount Sinai	Research Coordinator
Robin Fallegger	robin.fallegger@uni-heidelberg.de	Heidelberg University	Fellow
Melissa A Farrow	melissa.a.farrow@vanderbilt.edu	Vanderbilt University	Project Manager
Michael Ferkowicz	mferkow@iu.edu	Indiana University	Co-Investigator
Damian Fermin	dfermin@umich.edu	University of Michigan	Study staff
Derek M. Fine	dfine1@jhmi.edu	Johns Hopkins University	Biopsy Operator

Siobhan M. Flanagan, MD	flan0118@umn.edu	University of Minnesota	Biopsy Operator
Agnes B. Fogo	Agnes.fogo@vumc.org	Vanderbilt University	Co-Investigator,Pathologist
Monica L. Fox	mfox@nkfi.org	University of Illinois, Chicago	Patient Partner
Renee Frey	frenee@med.umich.edu	University of Michigan	Other
Anne Froment	anne_froment@med.unc.edu	University of North Carolina	Research Coordinator
Ron C. Gaba	rgaba@uic.edu	University of Illinois, Chicago	Co-Investigator,Biopsy Operator
Crystal A Gadegbeku	gadegbc@ccf.org	Cleveland Clinic	Co-Investigator
Lili Gai	lili.gai@mssm.edu	Mount Sinai	Other
Manoj Kumar Galla	manojkumargalla@ufl.edu	University of Florida	Other
Griselda Gamez	ggamez@arizona.edu	University of Arizona	Research Coordinator
Joseph P. Gaut	Jpgaut@wustl.edu	Washington University St. Louis	Pathologist
Kifle Gebre	kifle.gebre@bmc.org	Boston Medical Center	Other
Nils Gehlenborg	nils@hms.harvard.edu	Harvard University	Co-Investigator
Ann Gentry	ahgentry@gmail.com	University of Minnesota	Patient Partner
Molly C Geraghty	molly.geraghty@bmc.org	Boston Medical Center	Pathologist
Reetika Ghag	g.reetika@wustl.edu	Washington University St. Louis	Other
Matthew Gilliam	MJG136@pitt.edu	University of Pittsburgh	Other
Brandon Ginley		Other	Other
Debora Gisch	dgisch@iu.edu	Indiana University	Co-Investigator,Fellow
Ronald E. Gordon	Ronald.Gordon@mountsinai.org	Mount Sinai	Pathologist
Brittney L. Gorman	Brittney.gorman@pnnl.gov	Pacific Northwest National Laboratory	Study staff
Mark L Green	mark.green2@mssm.edu	Mount Sinai	Project Manager
Anna Greka	agreka@broadinstitute.org	Broad Institute	Other
Stephanie M. Grewenow	stephaniegrewenow@gmail.com	University of Washington	Other
Ritu Gupta	ritu.gupta@mountsinai.org	Mount Sinai	Pathologist
Bhupendra Kumar Gurung	gurungb@uthscsa.edu	University of Texas Health Science Center at San Antonio	Other
Leah Guthrie	lguthrie@berkeley.edu	Other	Other
Nir Hacohen	nhacohen@broadinstitute.org	Broad Institute	Principal Investigator
Samuel Haddad	samuel_haddad@med.unc.edu	University of North Carolina	Other
Daniel E. Hall	hallde@upmc.edu	University of Pittsburgh	Other
Jens Hansen	jens.hansen@mssm.edu	Mount Sinai	Co-Investigator
Tasma Harindhanavudhi	hari0049@umn.edu	University of Minnesota	Co-Investigator
Josh Hartley	jhartle@med.umich.edu	University of Michigan	Other

John Hartman	jrhartma@med.umich.edu	University of Michigan	Study staff
Lynda Hayashi	lyndahayashi@gmail.com	University of Washington	Patient Partner
Jonathan Haydak	jonathan.haydak@mssm.edu	Mount Sinai	Fellow,Patient Partner
John Cijiang He	cijiang.he@mssm.edu	Mount Sinai	Co-Investigator
Yongqun He	yongqunh@umich.edu	University of Michigan	Co-Investigator
S. Susan Hedayati	Saghar.Hedayati@stonybrookmedicine.edu	University of Texas Southwestern	Co-Investigator
Dori Henderson	hend0054@umn.edu	University of Minnesota	Project Manager
Joel M Henderson	jhender9@bidmc.harvard.edu	Boston Medical Center	Pathologist
Allen R Hendricks	allen.hendricks@utsouthwestern.edu	University of Texas Southwestern	Co-Investigator,Pathologist
Asari Henshaw	asari.henshaw@joslin.harvard.edu	Joslin Diabetes Center	Research Coordinator
Leal Herlitz	herlitl@ccf.org	Cleveland Clinic	Pathologist
Jeanine Hernandez	jherna49@jhu.edu	Johns Hopkins University	Research Coordinator
Bruce W. Herr II	bherr@iu.edu	Indiana University Bloomington	Project Manager,Other
Jonathan Himmelfarb, MD	jonathan.himmelfarb@mssm.edu	Mount Sinai	Principal Investigator
Jeffrey B. Hodgin	jhodgin@umich.edu	University of Michigan	Principal Investigator,Co-Investigator,Pathologist
Andrew N Hoofnagle	ahoof@uw.edu	University of Washington	Co-Investigator
Carol R. Horowitz MD, MPH	carol.horowitz@mssm.edu	Mount Sinai	Co-Investigator
Hsieh EWY	elena.hsieh@cuanschutz.edu	University of Colorado	Principal Investigator
Yuankai Huo	yuankai.huo@vanderbilt.edu	Vanderbilt University	Principal Investigator
Courtney Huynh	chuynh24@bu.edu	Boston Medical Center	Research Coordinator
Ravi Iyengar	ravi.iyengar@mssm.edu	Mount Sinai	Co-Investigator
Sanjay Jain	sanjayjain@wustl.edu	Washington University St. Louis	Principal Investigator,Other
Danielle Janosevic	djanosev@iu.edu	Indiana University	Principal Investigator
Andrew Janowczyk	andrew.r.janowczyk@emory.edu	Emory	Co-Investigator
Vivian Jeffers	jefferv@ccf.org	Cleveland Clinic	Research Coordinator
J. Ashley Jefferson	jashleyj@u.washington.edu	University of Washington	Co-Investigator,Biopsy Operator
Nichole M. Jefferson	nicholejeff@gmail.com	University of Washington	Patient Partner
J Charles Jennette	charles_jennette@med.unc.edu	University of North Carolina	Pathologist
Camille Johansen	Camille.Johansen@joslin.harvard.edu	Joslin Diabetes Center	Other
Stacey Jolly	JOLLYS@ccf.org	Cleveland Clinic	Other
Christopher J. Jones	cjjones@umn.edu	University of Minnesota	Co-Investigator,Biopsy Operator
	evelinj@uw.edu	University of Washington	Research Coordinator

Jennifer L. Jones	jjones@aakp.org	University of North Carolina	Patient Partner
Kiasha Jones, RN, BSN	jonesk48@ccf.org	Cleveland Clinic	Research Coordinator
Cienn N. Joyeux	ciennjoy@uw.edu	University of Washington	Other
Wenjun Ju	wenjunj@med.umich.edu	University of Michigan	Co-Investigator
Vijayakumar R Kakade	vijayakumar.kakade@yale.edu	Yale University	Principal Investigator
Dhatri Kakarla	dhatri_kakarla@med.unc.edu	University of North Carolina	Research Coordinator
Badra Kalil	bkalil1@jh.edu	Johns Hopkins University	Research Coordinator
Sanjeeva P. Kalva	Sanjeeva.Kalva@UTSouthwestern.edu	University of Texas Southwestern	Co-Investigator,Biopsy Operator
Rachel R. Kaspari	kasp0154@umn.edu	University of Minnesota	Research Coordinator
RAVINDER JEET KAUR	kaur.ravinder@mayo.edu	Mayo Clinic	Research Coordinator,Study staff
Madhurima Kaushal	kaushalm@wustl.edu	Washington University St. Louis	Project Manager,Study staff
Nicole Keefe	nicole_keefe@med.unc.edu	University of North Carolina	Biopsy Operator
Mark S. Keller	mark_keller@hms.harvard.edu	Harvard University	Other
Sara S. Kelley	sara_kelley@med.unc.edu	University of North Carolina	Research Coordinator
John A. Kellum	kellum@pitt.edu	University of Pittsburgh	Other
K. J. Kelly	kajkelly@iu.edu	Indiana University	Co-Investigator
Tanika N. Kelly	tkelly5@uic.edu	University of Illinois, Chicago	Co-Investigator
Candice A Kent	candice.kent@yale.edu	Yale University	Study staff,Other
Asra Kermani MD	asra.kermani@utsouthwestern.edu	University of Texas Southwestern	Other
Taesoo Kim	tkim2@arizona.edu	University of Arizona	Co-Investigator
Krzysztof Kiryluk	kk473@cumc.columbia.edu	Columbia University	Principal Investigator
Susan Klett	susan@sgklett.com	University of Minnesota	Patient Partner
Richard A. Knight	richardknight.aakp@gmail.com	University of Washington	Patient Partner
Amanda Knoten	aknoten@wustl.edu	Washington University St. Louis	Other
Gina Koch	gina.koch@mssm.edu	Mount Sinai	Research Coordinator
Robert Koewler	rob.koewler@gmail.com	Other	Patient Partner,Other
Patricia Kovatch	patricia.kovatch@mssm.edu	Mount Sinai	Co-Investigator,Other
Matthias Kretzler	kretzler@umich.edu	University of Michigan	Principal Investigator
Angela R.S. Kruse	angela.kruse@vanderbilt.edu	Vanderbilt University	Study staff
Yogish C. Kudva	kudva.yogish@mayo.edu	Mayo Clinic	Principal Investigator
Aleksandra Kukla	kukla.aleksandra@mayo.edu	Mayo Clinic	Principal Investigator
Michael Kuperman	Kupermm@ccf.org	Cleveland Clinic	Pathologist
Corey Kurek	Reid.Corey@mayo.edu	Mayo Clinic	Research Coordinator
Leonie Küchenhoff	leonie.kuechenhoff@uni-heidelberg.de	Heidelberg University	Other
Asmita L	lagwankar@wustl.edu	Washington University St. Louis	Other

Blue B. Lake	blake@altoslabs.com	Other	Co-Investigator
Roy Lardenoije	r.lardenoije@tudelft.nl	Other	Co-Investigator
Astrid Larson	astrid.larson@bmc.org	Boston Medical Center	Research Coordinator
Brandon G Larson	brandon.larson@mssm.edu	Mount Sinai	Project Manager
James P. Lash	jplash@uic.edu	University of Illinois, Chicago	Principal Investigator
Zoltan G. Laszik	zoltan.laszik@ucsf.edu	University of California San Francisco	Principal Investigator
Khun Zaw Latt	latt@umich.edu	University of Michigan	Co-Investigator
Stewart H. Lecker	slecker@bidmc.harvard.edu	Beth Israel Deaconess Medical Center	Co-Investigator,Biopsy Operator
Dongwon Lee	dongwon.lee@childrens.harvard.edu	Boston Children's	Principal Investigator
Simon C. Lee	simoncraddock.lee@utsouthwestern.edu	University of Texas Southwestern	Other
Sora Lee	leesora@ad.unc.edu	University of North Carolina	Research Coordinator
Sean Lefferts	sean.lefferts@mssm.edu	Mount Sinai	Research Coordinator,Project Manager
Petra M. Leite	petra.leite@yale.edu	Yale University	Research Coordinator
Lilach O. Lerman	lerman.lilach@mayo.edu	Mayo Clinic	Co-Investigator
Melissa Leroux	lerouxm@uw.edu	University of Washington	Research Coordinator
Xiang Li	xl260@duke.edu	Duke University	Fellow
Chrysta C Lienczewski	boridley@umich.edu	University of Michigan	Other
Christine P Limonte	climonte@uw.edu	University of Washington	Co-Investigator,Fellow
Jia-Ren Lin	jia-ren_lin@hms.harvard.edu	Harvard University	Fellow
Lili Liu	liulili@med.umich.edu	University of Michigan	Co-Investigator,Study staff
Patricia Kovatch	yiyuan.liu@mssm.edu	Mount Sinai	Other
Daine Livingood	livingood.daineshieluh@mayo.edu	Mayo Clinic	Research Coordinator,Study staff
Harshit Lohaan	h.lohaan@ufl.edu	University of Florida	Other
Minxin Lu	minxinlu@bu.edu	Boston Medical Center	Co-Investigator,Study staff
Nicholas Lucarelli	nlucarelli@ufl.edu	University of Florida	Study staff
Jessica Lukowski	jessica.lukowski@pnnl.gov	Pacific Northwest National Laboratory	Other
Dawn M. Lum	lumd@uw.edu	University of Washington	Research Coordinator
Brendon Lutnick		Other	Other
Shihong Ma	shihong.ma@utsouthwestern.edu	University of Texas Southwestern	Research Coordinator
Sisi Ma	sisima@umn.edu	University of Minnesota	Co-Investigator
Anant Madabhushi	anantm@emory.edu	Emory	Principal Investigator
Shana Maikhor	Shana.Maikhor@bmc.org	Boston Medical Center	Other

Soumya Maity	maity@uthscsa.edu	University of Texas Health Science Center at San Antonio	Co-Investigator
Mallory Mandel	Mallory.Mandel@childrens.harvard.edu	Joslin Diabetes Center	Study staff
Iyad S. Mansour	iyadmansour@arizona.edu	University of Arizona	Co-Investigator
Weiguang Mao	wmao@flatironinstitute.org	Princeton University	Other
Laura H. Mariani	lmariani@umich.edu	University of Michigan	Co-Investigator
Marina Markovic	markovm@ccf.org	Cleveland Clinic	Research Coordinator
Nicole Marquez	nmarquez1@arizona.edu	University of Arizona	Research Coordinator
Jamie L. Marshall	jmarshal@broadinstitute.org	Broad Institute	Other
Meredith C McAdams	meredith.mcadams@UTSouthwestern.edu	University of Texas Southwestern	Co-Investigator
Robyn L. McClelland	rmcclell@uw.edu	University of Washington	Co-Investigator
Phillip J. McCown	pjlmac@umich.edu	University of Michigan	Study staff
Michelle L. McGowan	mcgowan.michelle2@mayo.edu	Mayo Clinic	Co-Investigator
Gearoid Michael McMahon	gmmcmahon@bwh.harvard.edu	Brigham and Women's Hospital	Co-Investigator,Biopsy Operator
Amy McMurray	amy.mcmurray@wustl.edu	Washington University St. Louis	Research Coordinator
Karla Mehl	km3246@cumc.columbia.edu	Columbia University	Other
Kristin Meliambro	kristin.meliambro@mssm.edu	Mount Sinai	Principal Investigator
Ricardo Melo Ferreira	rimelof@iu.edu	Indiana University	Co-Investigator
Katherine Mendoza	katherinemendoza@arizona.edu	University of Arizona	Research Coordinator
Steven Menez	smenez1@jh.edu	Johns Hopkins University	Co-Investigator
Rajasree Menon	rajmenon@umich.edu	University of Michigan	Co-Investigator
Ece Meram	meram003@umn.edu	University of Minnesota	Biopsy Operator
Natalie Meza	nmeza1@uic.edu	University of Illinois, Chicago	Research Coordinator
Lukasz G. Migas	l.g.migas@tudelft.nl	Other	Study staff
Chloe E. Miller	miller.chloe4@mayo.edu	Mayo Clinic	Research Coordinator
R. Tyler Miller	tyler.miller@utsouthwestern.edu	University of Texas Southwestern	Co-Investigator
Sayat Mimar	sayat.mimar@ufl.edu	University of Florida	Other
Brittany C Minor	bminor@wustl.edu	Washington University St. Louis	Study staff
Priya Mody	priya_mody@med.unc.edu	University of North Carolina	Biopsy Operator
Gilbert W. Moeckel	gilbert.moeckel@yale.edu	Yale University	Pathologist
Moledina DG	dennis.moledina@yale.edu	Yale University	Co-Investigator
Jenny Molina-Guzman	Jenny.Molina-Guzman@joslin.harvard.edu	Joslin Diabetes Center	Study staff
Jose M Monroy-Trujillo	jmonroy2@jhmi.edu	Johns Hopkins University	Co-Investigator,Biopsy Operator

Alexander Morales	Amorale7@bidmc.Harvard.edu	Beth Israel Deaconess Medical Center	Biopsy Operator
Vanessa Moreno	vanessa.moreno@unc.edu	University of North Carolina	Co-Investigator,Pathologist
Amy K. Mottl	amy_mottl@med.unc.edu	University of North Carolina	Principal Investigator
Keyvona Moultrie	keyvonam@bu.edu	Boston Medical Center	Other
Tariq Mukatash	Tariq.Mukatash@ucsf.edu	University of California San Francisco	Other
Dane Munar	dane.munar@ucsf.edu	University of California San Francisco	Research Coordinator
Raghavan Murugan	muruganr@upmc.edu	University of Pittsburgh	Principal Investigator
Patrick H. Nachman	pnachman@umn.edu	University of Minnesota	Principal Investigator
Girish N Nadkarni	girish.nadkarni@mountsinai.org	Mount Sinai	Principal Investigator
Ahmed Naglah	ahmed.naglah@ufl.edu	University of Florida	Study staff
Abhijit S. Naik	abhinaik@umich.edu	University of Michigan	Co-Investigator
Viji Nair	vijin@med.umich.edu	University of Michigan	Study staff
Behzad Najafian	najafian@uw.edu	University of Washington	Co-Investigator,Pathologist
Yunbi Nam	yunbinam@uw.edu	University of Washington	Other
Azuma Nanamatsu	ananamat@iu.edu	Indiana University	Fellow
Narasimhan, R.	rnarasimhan3@mgb.org	Boston Medical Center	Pathologist
Nhung Nguyen	ngleena@uw.edu	University of Washington	Project Manager,Study staff
Gerald Nwanne	Nwanne@wustl.edu	Washington University St. Louis	Study staff
Charles O'Malley, MD	omallec@ccf.org	Cleveland Clinic	Co-Investigator,Biopsy Operator
John F. O'Toole	otoolej@ccf.org	Cleveland Clinic	Principal Investigator
Fernanda Ochoa Toro	ferochoa@ad.unc.edu	University of North Carolina	Research Coordinator
George (Holt) Oliver	george.oliver@phhs.org	Parkland Health and Hospital System	Other
Oluwatosin Oluwole	oluwo006@umn.edu	University of Minnesota	Research Coordinator
Ingrid F Onul	ingrid.onul@bmc.org	Boston Medical Center	Research Coordinator
Edgar A. Otto	eotto@umich.edu	University of Michigan	Co-Investigator
Paul M. Palevsky	palevsky@pitt.edu	University of Pittsburgh	Principal Investigator
Ellen Palmer	elp76@case.edu	Cleveland Clinic	Other
Annapurna Pamreddy	pamreddy@uthscsa.edu	University of Texas Health Science Center at San Antonio	Other
Chirag R. Parikh	chirag.parikh@jhmi.edu	Johns Hopkins University	Principal Investigator
Samir V Parikh	samir.parikh@osumc.edu	Ohio State University	Co-Investigator
Christopher Park	cpark3@nephrology.washington.edu	University of Washington	Other

Harold Park	Harold.park@utsouthwestern.edu	University of Texas Southwestern	Other
Ljiljana Paša-Tolić	ljiljana.pasatolic@pnnl.gov	Pacific Northwest National Laboratory	Co-Investigator
Jiten Patel	Jiten.Patel@UTSouthwestern.edu	University of Texas Southwestern	Co-Investigator
Marissa Patel	marissa.patel@mssm.edu	Mount Sinai	Research Coordinator
Boris S. Patlis	boris.patlis@utsouthwestern.edu	University of Texas Southwestern	Research Coordinator,Other
Anindya S. Paul	Anindya.Paul@medicine.ufl.edu	University of Florida	Study staff
Jimmy Phuong	jphuong@uw.edu	University of Washington	Study staff
Anil Pillai	Anil.pillai@utsouthwestern.edu	University of Texas Southwestern	Other
Roy Pinkeney	20shuvee11@gmail.com	Other	Patient Partner,Other
Alexa Plisiewicz	alexa.plisiewicz@mssm.edu	University of Washington	Project Manager
Emilio D Poggio	poggioe@ccf.org	Cleveland Clinic	Principal Investigator,Co-Investigator
Ari Pollack	ari.pollack@seattlechildrens.org	Other	Principal Investigator
Pottumarthi V Prasad	p-prasad2@northwestern.edu	Northwestern University	Co-Investigator
Laura Pyle	lpyle@uw.edu	University of Washington	Co-Investigator
Ellen M. Quardokus	ellenmq@iu.edu	Indiana University Bloomington	Other
Timothy D. Quinn	timothy.quinn@mountsinai.org	Mount Sinai	Other
Arabela Quiroga	aquiro23@uic.edu	University of Illinois, Chicago	Research Coordinator
Salma Rabi	srabi@umn.edu	University of Minnesota	Research Coordinator
NAGARJUNACHARY RAGI	ragi@uthscsa.edu	University of Texas Health Science Center at San Antonio	Study staff
Parmjeet Randhawa	randhawapa@upmc.edu	University of Pittsburgh	Pathologist
Teresa Randle	randlet@ccf.org	Cleveland Clinic	Research Coordinator
Tejas Rao	tejas.rao@mssm.edu	Mount Sinai	Study staff
Via Rao	rao00105@umn.edu	University of Minnesota	Research Coordinator
Michael Rauchman	mrauchma@wustl.edu	Washington University St. Louis	Principal Investigator
Nicolas J Rauwolf	rauwo004@umn.edu	University of Minnesota	Research Coordinator
Rebecca Reamy	rlreamy@umich.edu	University of Michigan	Study staff
Elizabeth G. Record	recorde@iu.edu	Indiana University Bloomington	Research Coordinator
Devona Redmond	dgamb2@uic.edu	University of Illinois, Chicago	Study staff,Other
Stephanie Reinert	reinert@wustl.edu	Washington University St. Louis	Other
Joseph Reis	joseph.reis@seattlechildrens.org	University of Washington	Co-Investigator
Helmut Rennke	hrennke@bwh.harvard.edu	Brigham and Women's Hospital	Other
Amada Renteria	Amada@uic.edu	University of Illinois, Chicago	Research Coordinator

Kasra A Rezaei	Krezaei@uw.edu	University of Washington	Co-Investigator,Other
Rosamond Rhodes	rosamond.rhodes@mssm.edu	Other	Co-Investigator
Ana C. Ricardo	aricar2@uic.edu	University of Illinois, Chicago	Principal Investigator
Marcelino Rivera	maeriver@iu.edu	Indiana University	Co-Investigator
Glenda V. Roberts	glenda.roberts@mssm.edu	Mount Sinai	Patient Partner
Cassianne Robinson-Cohen	cassianne.robinson-cohen@vanderbilt.edu	Vanderbilt University	Co-Investigator
Elizabeth A. Rogers	earogers@umn.edu	University of Minnesota	Co-Investigator
Florencia A. Rojas-Miguez	florencia.rojas-miguez@bmc.org	Boston Medical Center	Other
Sophia H. Rosan	Sophia.Rosan@bmc.org	Boston Medical Center	Research Coordinator
Rosas, Sylvia E.	sylvia.rosas@joslin.harvard.edu	Joslin Diabetes Center	Principal Investigator
Michael P. Rose	rosemi@med.umich.edu	University of Michigan	Study staff
Seymour Rosen	srosen@bidmc.harvard.edu	Harvard University	Pathologist
Avi Z. Rosenberg	arosen34@jh.edu	Johns Hopkins University	Pathologist
Michael S. Rosenberg	rosen011@umn.edu	University of Minnesota	Co-Investigator
Matthew R. Rosengart	mrr18@pitt.edu	University of Pittsburgh	Principal Investigator
Brad H. Rovin	rovin.1@osu.edu	Ohio State University	Principal Investigator
Neil Roy	neil.roy@joslin.harvard.edu	Joslin Diabetes Center	Research Coordinator
Prabir Roy-Chaudhury	prabir@email.unc.edu	University of North Carolina	Co-Investigator
Melissa D. Rubinsky	melissa.rubinsky@joslin.harvard.edu	Joslin Diabetes Center	Study staff,Other
Angela R. Sabo	saboa@iu.edu	Indiana University	Study staff
Tami Sadusky	saduskyts@gmail.com	University of Washington	Patient Partner
Julio Saez-Rodriguez	saez@ebi.ac.uk	Heidelberg University	Principal Investigator
Sami Safadi	safad002@umn.edu	University of Minnesota	Co-Investigator
Imane H. Samari	imane.samari@joslin.harvard.edu	Joslin Diabetes Center	Research Coordinator
Ana Celina Sanora	celinasanora@arizona.edu	University of Arizona	Research Coordinator
Sandro Santagata	ssantagata@bwh.harvard.edu	Harvard University	Principal Investigator
Pinaki Sarder	pinaki.sarder@ufl.edu	University of Florida	Co-Investigator
Natalya Sarkisova	nasark@uw.edu	University of Washington	Project Manager
Minnie M Sarwal	Minnie.sarwal@ucsf.edu	University of California San Francisco	Principal Investigator
John Saul	jbsaul@mac.com	Other	Patient Partner,Other
Milda R. Saunders	msaunders@uchicago.edu	Other	Co-Investigator
Jennifer A. Schaub	schaubj@med.umich.edu	University of Michigan	Co-Investigator
IM Schmidt	ischmidt@bu.edu	Boston Medical Center	Co-Investigator
Raymond Scott	raymondscott68@gmail.com	University of Arizona	Patient Partner
Aaron Scroggins	ascroggi@uic.edu	University of Illinois, Chicago	Other
Rachel S. G. Sealfon	rsealfon@flatironinstitute.org	Princeton University	Co-Investigator
John R. Sedor	sedorj@ccf.org	Cleveland Clinic	Principal Investigator

Dianna Sendrey, RN, BSN	sendred2@ccf.org	Cleveland Clinic	Research Coordinator
Maninderpal S. Sethi	sethim@uw.edu	University of Washington	Co-Investigator
Sanjeev Sethi	sethi.sanjeev@mayo.edu	Mayo Clinic	Pathologist
Suman Setty	ssetty@uic.edu	University of Illinois, Chicago	Pathologist
Sonya Shah	sshah115@jhu.edu	Johns Hopkins University	Research Coordinator
Saad Mohammed Shariff	saad_shariff@med.unc.edu	University of North Carolina	Biopsy Operator
Kumar Sharma	sharmak3@uthscsa.edu	University of Texas Health Science Center at San Antonio	Principal Investigator
Sandeep Sharma	sharm340@umn.edu	University of Minnesota	Co-Investigator,Biopsy Operator
Melissa M. Shaw	melissa.m.shaw@yale.edu	Yale University	Research Coordinator,Study staff
Tara K Sigdel	tara.sigdel@ucsf.edu	University of California San Francisco	Co-Investigator
Kim Silva	ksilva4@uic.edu	University of Illinois, Chicago	Research Coordinator,Project Manager
Paolo S. Silva	paoloantonio.silva@joslin.harvard.edu	Joslin Diabetes Center	Co-Investigator
Emnet Sisay	es8173@princeton.edu	Princeton University	Study staff
Cathy Smith	smithcat@umich.edu	University of Michigan	Study staff
Kelly D. Smith	kelsmith@uw.edu	University of Washington	Co-Investigator,Pathologist
Jaime Snyder	jas1208@uw.edu	University of Washington	Co-Investigator
Michelle L. Snyder RN	snyde003@umn.edu	University of Minnesota	Research Coordinator
Mohammad A. Sohail	masohail@iu.edu	Indiana University	Fellow
Ksenia Sokolova	sokolova@princeton.edu	Princeton University	Other
Kassandra Spates-Harden	spatesk2@ccf.org	Cleveland Clinic	Patient Partner
C. John Sperati	jsperati@jhmi.edu	Johns Hopkins University	Biopsy Operator
Jeffrey M. Spraggins	jeff.spraggins@vanderbilt.edu	Vanderbilt University	Principal Investigator
Anand Srivastava	asrivast@uic.edu	University of Illinois, Chicago	Principal Investigator,Co-Investigator,Other
Daniel Stalbow	daniel.stalbow@mssm.edu	Mount Sinai	Biopsy Operator
Jennifer Stashevsky	jstashev@iupui.edu	Indiana University	Study staff
Anna Kate Stawicki	anna.stawicki@joslin.harvard.edu	Joslin Diabetes Center	Study staff
Becky Steck	roesch@med.umich.edu	University of Michigan	Other
Isaac E Stillman	Isaac.Stillman@mountsinai.org	Mount Sinai	Pathologist

Christy Stutzke	dzinri@aol.com	University of Washington	Patient Partner
Lalita Subramanian	lsub@umich.edu	University of Michigan	Project Manager
Jennifer K. Sun, MD, MPH	jennifer.sun@joslin.harvard.edu	Joslin Diabetes Center	Co-Investigator
Sandhya Sundar Rajan	ssandhya@med.unc.edu	University of North Carolina	Research Coordinator,Study staff
Timothy A. Sutton	tsutton2@iu.edu	Indiana University	Co-Investigator
Jonathan J Taliercio	talierj@ccf.org	Cleveland Clinic	Co-Investigator
Roderick Tan	tanrj@upmc.edu	University of Pittsburgh	Other
Jovan Tanevski	jovan.tanevski@uni-heidelberg.de	Heidelberg University	Co-Investigator
Michael Tanious	mtanio2@uic.edu	University of Illinois, Chicago	Biopsy Operator
thajudeen b	bijint@deptofmed.arizona.edu	University of Arizona	Co-Investigator
Heather Thiessen Philbrook	hthiess1@jh.edu	Johns Hopkins University	Co-Investigator
Joshua M. Thurman	joshua.thurman@cuanschutz.edu	University of Colorado	Principal Investigator
Joji Tokita	joji.tokita@mssm.edu	Mount Sinai	Co-Investigator,Biopsy Operator
Kalie L. Tommerdahl	ktomme@uw.edu	University of Washington	Co-Investigator
Jose R. Torrealba	jose.torrealba@utsouthwestern.edu	University of Texas Southwestern	Pathologist
Robert D Toto	Robert.Toto@UTSouthwestern.edu	University of Texas Southwestern	Principal Investigator
Haneen Tout	haneen@umich.edu	University of Michigan	Other
Olga G Troyanskaya	ogt@princeton.edu	Princeton University	Principal Investigator
Jeffrey M Turner	jeffrey.turner@yale.edu	Yale University	Biopsy Operator
Katherine R. Tuttle	katherine.tuttle@providence.org	University of Washington	Co-Investigator
Ugochukwu Ugwuowo	ugochukwu.ugwuowo@yale.edu	Yale University	Research Coordinator
Ashish Upadhyay	ashish.upadhyay@bmc.org	Boston Medical Center	Co-Investigator
Rachel Ustoyev	rachel.ustoyev@mssm.edu	Mount Sinai	Research Coordinator
M. Todd Valerius	todd@valeriuslab.org	Brigham and Women's Hospital	Co-Investigator
Raf Van de Plas	raf.vandeplas@tudelft.nl	Other	Principal Investigator
Heidi L. Vandyk	heidi.vandyk@pnnl.gov	Pacific Northwest National Laboratory	Study staff
German varela	gv2302@cumc.columbia.edu	Columbia University	Research Coordinator
Miguel A. Vazquez	miguel.vazquez@utsouthwestern.edu	University of Texas Southwestern	Principal Investigator
Dusan Velickovic	dusan.velickovic@pnnl.gov	Pacific Northwest National Laboratory	Co-Investigator
Marija Velickovic	marija.velickovic@pnnl.gov	Pacific Northwest National Laboratory	Study staff

manjeri venkatachalam	venkatachal@uthscsa.edu	University of Texas Health Science Center at San Antonio	Co-Investigator,Pathologist
Abraham Verdoes	averdoes@iu.edu	Indiana University	Project Manager
Ashish Verma	ashverma@bu.edu	Boston Medical Center	Co-Investigator
Angela M. Victoria-Castro	angela.victoria-castro@yale.edu	Yale University	Research Coordinator
Anitha Vijayan	avijayan@wustl.edu	Washington University St. Louis	Co-Investigator
Carissa Vinovskis	Carissa.Vinovskis@childrenscolorado.org	University of Colorado	Other
Tina Vita	vitatm@upmc.edu	University of Pittsburgh	Research Coordinator
Sushrut S. Waikar	swaikar@bu.edu	Boston Medical Center	Principal Investigator
N/A	wjwaliggo@gmail.com	Joslin Diabetes Center	Other
Ashley R. Wang	awang78@jhu.edu	Johns Hopkins University	Research Coordinator
Bangchen Wang	bangchen.wang@duke.edu	Duke University	Pathologist
Nancy Wang	zhengnan.wang@utsouthwestern.edu	University of Texas Southwestern	Research Coordinator
Ruikang Wang	wangrk@uw.edu	University of Washington	Co-Investigator,Other
Artit Wangperawong	artitw@uw.edu	University of Washington	Study staff
Stephen C Ward	stephen.ward@mountsinai.org	Mount Sinai	Pathologist
Curtis Warfield	chwarf01@gmail.com	Indiana University	Patient Partner
Astrid Weins	aweins@bwh.harvard.edu	Brigham and Women's Hospital	Pathologist
Julia A. Welch	Julia.Welch@joslin.harvard.edu	Joslin Diabetes Center	Study staff,Other
Natasha Wen	yu.wen@utsouthwestern.edu	University of Texas Southwestern	Other
Yumeng Wen	ywen14@jhmi.edu	Johns Hopkins University	Co-Investigator,Fellow
Aaron Wightman	aaron.wightman@seattlechildrens.org	University of Washington	Co-Investigator
Adam Wilcox	abwilcox@uw.edu	University of Washington	Other
James C. Williams, Jr.	jwillia3@iu.edu	Indiana University	Principal Investigator
Kayleen Williams	kmfw@u.washington.edu	University of Washington	Other
Mark E. Williams MD	mark.williams@joslin.harvard.edu	Beth Israel Deaconess Medical Center	Co-Investigator
F. Perry Wilson	francis.p.wilson@yale.edu	Yale University	Principal Investigator,Co-Investigator
Seth Winfree	winfrees@iu.edu	Other	Co-Investigator
James Winters	wintersj4@upmc.edu	University of Pittsburgh	Other
Stephanie Wofford	sdwillia@iu.edu	Indiana University	Research Coordinator
Susan M. Wolf	swolf@umn.edu	University of Minnesota	Co-Investigator
Aaron Wong	awong@flatironinstitute.org	Princeton University	Other
Gregory Woodhead	gregorywoodhead@arizona.edu	University of Arizona	Co-Investigator,Biopsy Operator
Devin M. Wright	devmwrig@iu.edu	Indiana University	Project Manager
Zach Wright	zwright@umich.edu	University of Michigan	Other
Zoe Wright	wrig1316@umn.edu	University of Minnesota	Research Coordinator

Julia Wrobel	julia.wrobel@cuanschutz.edu	University of Colorado	Co-Investigator
Alan Xu	axu9@jhu.edu	Johns Hopkins University	Other
Sophia Xu	sxu80@jh.edu	Johns Hopkins University	Research Coordinator
Pranav Yadati	pranav.yadati@bmc.org	Boston Medical Center	Research Coordinator
Johnson Yang	yang2605@umn.edu	University of Minnesota	Research Coordinator
Hongping Ye	yeh1@uthscsa.edu	University of Texas Health Science Center at San Antonio	Other
Bessie A. Young, MD, MPH	youngb@uw.edu	University of Washington	Co-Investigator
Guanghao Yu, BA	guanghao.yu@bmc.org	Boston Medical Center	Research Coordinator
Samuel Mon-Wei Yu	mon-wei.yu@mountsinai.org	Mount Sinai	Study staff
Gabriel Zeinoun	gabriel.zeinoun@joslin.harvard.edu	Joslin Diabetes Center	Research Coordinator
Evan M. Zeitler	evan.zeitler@unchealth.unc.edu	University of North Carolina	Co-Investigator
Bo Zhang	bzhang22@wustl.edu	Washington University St. Louis	Study staff
Guanshi Zhang	zhangg3@uthscsa.edu	University of Texas Health Science Center at San Antonio	Co-Investigator
Kun Zhang	k4zhang@ucsd.edu	University of California San Diego	Co-Investigator
Shiqi Zhang	zhangs2@uthscsa.edu	University of Texas Health Science Center at San Antonio	Other
Yi Zhang	yizhang1@uw.edu	Harvard University	Fellow
Yan Zhou	yanzhou@bu.edu	Boston Medical Center	Fellow
